# Supplementary material for: Time-series transcriptome analysis identified differentially expressed genes in broiler chicken infected with mixed Eimeria species
Source: Front Genet. 2022 Aug 8;13:886781. doi: 10.3389/fgene.2022.886781 (PMC9393255; doi:10.3389/fgene.2022.886781)
Supplement: Supplementary file 2 [file DataSheet1.ZIP › 7dpi_KEGG.Gsea.1625115961950/index.html]

Index for xtools.gsea.Gsea 21070\_7dpi\_KEGG.Gsea.1625115961950

### GSEA Report for Dataset TMM\_7dpi\_gct\_format

#### Enrichment in phenotype: **1 (5 samples)**

- 51 / 186 gene sets are upregulated in phenotype **1**- 15 gene sets are significant at FDR < 25%- 12 gene sets are significantly enriched at nominal pvalue < 1%- 13 gene sets are significantly enriched at nominal pvalue < 5%- Snapshot of enrichment results- Detailed enrichment results in html format- Detailed enrichment results in TSV format (tab delimited text)- Guide to interpret results

#### Enrichment in phenotype: **0 (3 samples)**

- 135 / 186 gene sets are upregulated in phenotype **0**- 51 gene sets are significantly enriched at FDR < 25%- 21 gene sets are significantly enriched at nominal pvalue < 1%- 41 gene sets are significantly enriched at nominal pvalue < 5%- Snapshot of enrichment results- Detailed enrichment results in html format- Detailed enrichment results in TSV format (tab delimited text)- Guide to interpret results

#### Dataset details

- The dataset has 12011 features (genes)- No probe set => gene symbol collapsing was requested, so all 12011 features were used

#### Gene set details

- Gene set size filters (min=3, max=1000) resulted in filtering out 0 / 186 gene sets- The remaining 186 gene sets were used in the analysis- List of gene sets used and their sizes (restricted to features in the specified dataset)

#### Gene markers for the **1** *versus* **0** comparison

- The dataset has 12011 features (genes)- # of markers for phenotype **1**: 4040 (33.6% ) with correlation area 27.3%- # of markers for phenotype **0**: 7971 (66.4% ) with correlation area 72.7%- Detailed rank ordered gene list for all features in the dataset- Heat map and gene list correlation  profile for all features in the dataset

#### Global statistics and plots

- Plot of p-values *vs.* NES- Global ES histogram

#### Other

- Parameters used for this analysis

#### Comments

- There were duplicate row identifiers in the specified dataset. One id was arbitarilly choosen. Details are below
  Generally, this is OK, but if you want to avoid this automagic, edit your dataset so that all row ids are unique
  # of row ids in original dataset: 12076
  # of row UNIQUE ids in original dataset: 12011
  # The duplicates were
  PUSL1
  RHOGL
  LOC772071
  PTTG1IP
  01-Mar
  AQR
  HEBP1
  LOXL4
  CERS3
  PLA2G2E
  ALG9
  PARP14
  TGM2
  B4GALT3
  HPS5
  KCTD12
  P3H3
  CCL4
  HIST2H3A
  LOC100857439
  SYNJ2BP
  IGSF9
  HIST2H3A
  LOC100857439
  DHRS11
  RASAL3
  HIST1H4D
  HIST1H4B
  GOLPH3L
  ZMYM2
  LOC776067
  AOC3
  HIST1H4D
  HIST1H4B
  WISP2
  DGUOK
  MAT2A
  RABGAP1L
  IFI35
  HIST1H4D
  HIST1H4B
  HIST1H2A4
  LOC416147
  SUV39H1
  FOXK2
  HIST1H2A4
  FAM83D
  MICA
  HIST1H4D
  HIST1H4B
  HIST2H3A
  LOC100857439
  HIST1H2A4
  HIST1H2A4
  LOC771880
  RAP1GAP2
  HIST1H4D
  HIST1H4B
  GOLPH3L
  GZF1
  HIST1H4D
  HIST1H4B
  HIST1H2A4
  GDAP1L1
  RGS3- Timestamp used as random seed: 1625115966189

---

Report: 21070\_7dpi\_KEGG.Gsea.1625115961950.rpt   by user: user

xtools.gsea.Gsea [Thu, Jul 1, '21 2 PM 6]

Website: www.gsea-msigdb.org/gsea
Questions & Suggestions: Contact page
